# Household economic burden and outcomes of patients with schizophrenia after being unlocked and treated in rural China

**DOI:** 10.1017/S2045796019000775

**Published:** 2019-12-16

**Authors:** L. Xu, T. Xu, W. Tan, B. Yan, D. Wang, H. Li, Y. Lin, K. Li, H. Wen, X. Qin, X. Sun, L. Guan, J.K. Bass, H. Ma, X. Yu

**Affiliations:** 1 Peking University Sixth Hospital, Peking University Institute of Mental Health, NHC Key Laboratory of Mental Health (Peking University), National Clinical Research Center for Mental Disorders (Peking University Sixth Hospital), CN 100191, China; 2 Guangdong General Hospital, Guangdong Mental Health Center, Guangzhou, Guangdong, CN, China; 3 Hebei Sixth People's Hospital, Heibei Institute of Mental Health, Baoding, Hebei, CN, China; 4 The Third People's Hospital of Mianyang, Sichuan Mental Health Center, Mianyang, Sichuan, CN, China; 5 Department of Prevention and Control, The Fourth People's Hospital of Chengdu, Chengdu, Sichuan, CN, China; 6 Department of Mental Health, Johns Hopkins Bloomberg School of Public Health, Baltimore, MD, USA

**Keywords:** Economic issues, families, health service research, risk factors, schizophrenia

## Abstract

**Aims:**

Patients with severe mental disorders in low-resource settings have limited access to services, resulting in overwhelming caregiving burden for families. In extreme cases, this has led to the long-term restraining of patients in their homes. China underwent a nationwide initiative to unlock patients and provide continued treatment. This study aims to quantify household economic burden in families after unlocking and treatment, and to identify factors associated with increased burden due to schizophrenia.

**Methods:**

A total of 264 subjects were enrolled from three geographically diverse provinces in 2012. Subjects were patients with schizophrenia who were previously put under restraints and had participated in the ‘unlocking and treatment’ intervention. The primary outcome was the current household economic burden, obtained from past year financial information collected through on-site interview. Patient disease characteristics, treatment, outcomes and family caregiving burden were collected as well. Univariate and multivariate linear regression were used to construct risk factor models for indirect economic burden.

**Results:**

After participating in the intervention, 85% of patients continued to receive mental health services, 70% used medication as prescribed and 80% were never relocked. Family members reported significantly decreased caregiving burden after receiving the intervention. Mean direct and indirect household economic burdens were CNY963 (US$31.7) and CNY11 724 (US$1670) per year, respectively, while family total income was on average CNY12 108 (US$1913) per year. Greater disease severity and poorer patient psychosocial function at time of study were found to be independent factors related to increased indirect burden.

**Conclusions:**

The ‘unlocking and treatment’ intervention has improved the lives of patients and families. Indirect burden due to disease is still a major economic issue that needs to be addressed, potentially through improving treatment and patient functioning. Our findings contribute to the unravelling and eventual elimination of chronic restraining of mentally ill patients in low-resource settings.

## Introduction

Schizophrenia is a chronic debilitating disease affecting 0.5–1% of the general population (Simeone *et al*., 2015). In low-resource settings, barriers such as fragmented mental health care systems and lack of trained providers have resulted in a persistent unmet need for care (Saraceno *et al*., 2007), often resulting in family members taking on greater caregiving responsibilities. However, poverty, scarcity of treatment options and lack of awareness of patient rights in these communities have perpetuated the practice of physically restraining patients within their homes. This is a global phenomenon that has been described in Pakistan (Malik, 2001), Ghana (Read *et al*., 2009), Somalia (Ndetei and Mbwayo, 2010), Indonesia (Minas and Diatri, 2008; Puteh *et al*., 2011) and Ethiopia (Asher *et al*., 2017), however its extent has not been systematically assessed. Of these countries, only Indonesia has reported intervention efforts (Puteh *et al*., 2011).

China introduced a national initiative to scale up community mental health services in 2005, known as the ‘686’ Program, details of which are reported elsewhere (Liu *et al*., 2011; Good and Good, 2012; Ma, 2012; Guan *et al*., 2015). Through this initiative, the presence of long-term restraining in rural communities was brought to the attention of policy makers. An ‘unlocking and treatment’ intervention was subsequently established, with components including active outreach to families, unlocking patients in the home, medical treatment following release and community follow-up. Initially, CNY700 per case per year (US$86 by 2005 conversion rates) was allocated for unlocking and treating patients. This was increased to CNY5000 per case per year (US$732 by 2008 conversion rates) in 2008. Funding covered home visits to unlock patients, onsite emergency psychiatric assessment and treatment, and hospitalisation following release.

A pilot study showed that the programme was successful in unlocking patients and improving the quality of life of both patients and caregivers (Good and Good, 2012; Guan *et al*., 2015). Despite initial success, new cases of restraining are continuously identified across the country, while a portion of those liberated are eventually put under restraints again in the community, indicating an urgent need to better understand underlying social-economic determinants of restraining patients.

In our previous study, 96% of families responded that financial difficulty was the main reason for restraint (Guan *et al*., 2015). A recent qualitative study in Indonesia also found that financial constraints were a barrier to care in this population (Laila *et al*., 2018). However, the extent of caregiver burden and financial difficulties have not been further evaluated, and whether such difficulties still exist after receiving the ‘unlocking and treatment’ intervention is of interest.

Apart from documenting outcomes following the ‘unlocking and treatment’ intervention, the present study aims, in particular, to quantify the current household economic burden due to schizophrenia and to identify independent factors associated with increased burden. Such information may contribute to improvement of the programme in China, as well as provide reference for low- and middle-income settings worldwide.

## Methods

### Procedure of the ‘unlocking and treatment’ intervention

The procedure of the ‘unlocking and treatment’ intervention is described elsewhere (Good and Good, 2012; Ma, 2012; Guan *et al*., 2015). In brief: The process began with a team consisting of a mental health personnel, community physician and neighbourhood committee member visiting the homes of those identified to be under restraints. After communicating benefits and patient rights, signed consent was asked for beginning the unlocking process. Following the release, patients were either hospitalised or enrolled directly in standard ‘686’ community services. Hospitalised patients also received standard ‘686’ services after discharge, which included monthly follow-up visits, subsidised medication and community-based rehabilitation. Health education and psychosocial support were provided throughout the process.

### Study design and participants

We implemented an observational study between July and September of 2012, with two major components: (1) a cross-sectional home survey to assess the patient, interview families and collect household financial data; and (2) a retrospective investigation of patient outcomes and caregiving burden through on-site assessment supplemented by patient records.

Patients fulfilling the following criteria were included: (1) was clinically diagnosed with schizophrenia using ICD-10 criteria; (2) aged between 18 and 60 years old; (3) had been subjected to physical restraints at home for at least 3 months; (4) was released between 1 January 2005 and 1 June 2012 through the ‘unlocking and treatment’ intervention, and immediately admitted to inpatient psychiatric services following release. Exclusion criteria were: (1) unable to identify a legal guardian; (2) caregiver unable to understand the informed consent or research questions.

### Subject recruitment

As the primary measurement of interest was economic burden due to disease, we calculated the sample size for the purpose of estimating this figure. Based on unpublished pilot data, the standard deviation for this variable was CNY8371. Sample size was estimated to be 270 using the following formula under a significance level of *α* = 0.05 and allowing a confidence interval width of CNY2000.
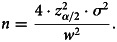


**Fig. 1.**
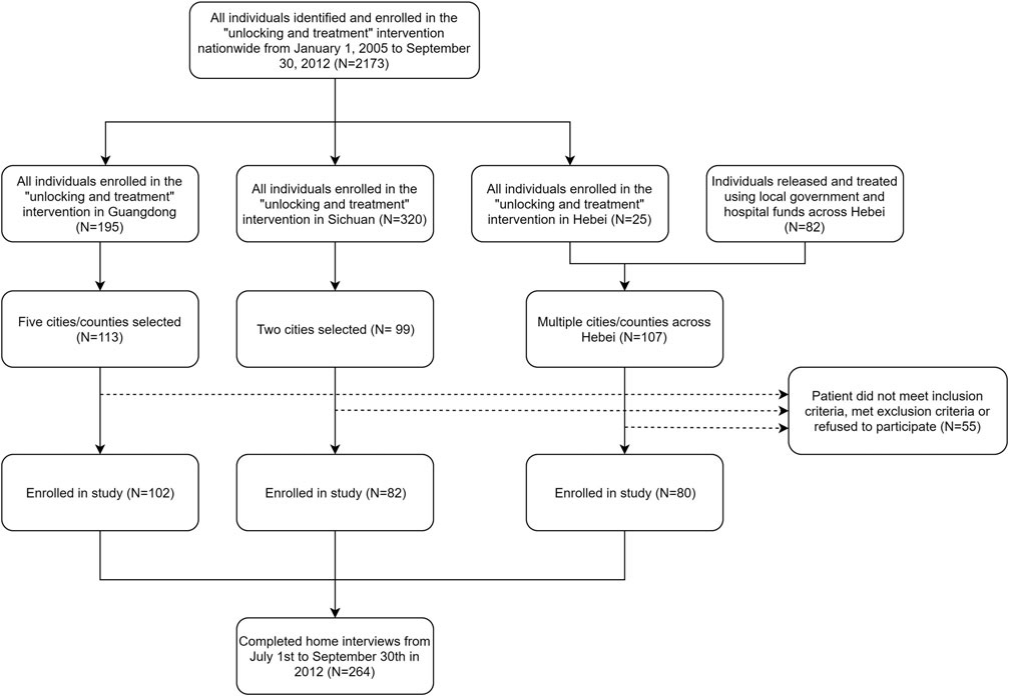
Procedure for sampling and enrollment.

To recruit a representative sample, we selected three geographically and economically diverse provinces: Guangdong (eastern area, high economic development), Hebei (middle area, medium economic development) and Sichuan (western area, relatively low economic development). These provinces had high numbers of ‘unlocked’ cases, allowing for adequate enrolment. Several counties in each province were selected as study sites based on two criteria: (1) the county had a public mental health institution, and (2) cases were concentrated within that county. Within the study sites, a total of 319 cases were identified (Fig. 1), of which 264 (83%) were included in the study.

### Data collection

Each study site established a team of 5–8 professionals from the city or provincial-level psychiatric hospitals, including psychiatrists, psychiatric nurses and community mental health service coordinators. Researchers underwent systematic training prior to beginning the study. A data collection form was designed for this study based on literature review, expert opinion, our conceptual framework and previous results (Guan *et al*., 2015). A pilot study was conducted to assess its applicability, and modifications were made accordingly. Data quality was maintained through self-monitoring by data collectors, supervision by coordinators at each study site and supervision from the central institution. Primary informants were mostly parents (60%), siblings (19%) and spouses (14%). For 49 out of 264 subjects (18.6%), two or more family caregivers provided information.

Data used in this study were gathered for two time points: when patients were first unlocked (T-unlocking) and the time of the interview (T-study). For T-study, investigators collected demographic information and data regarding the use of restraints during home visits. Clinical characteristics (length of disease, family history of mental disorders, illness severity, violent behaviour, psychosocial function, medication usage and perceived caregiving burden) were assessed by the psychiatrist based on interviews with families and patients, and further corroborated by medical and case records. Importantly, household economic data were collected during face-to-face interviews at T-study. For T-unlocking, most data were obtained from medical and case management records dated from when the patient was first unlocked, including clinical diagnosis, illness severity, violent behaviour, psychosocial function and medication usage. Perceived caregiving burden at time of unlocking was rated by retrospectively asking patient caregivers during the home visit in 2012.

### Diagnoses

As described previously (Guan *et al*., 2015), following unlocking and hospitalisation, a psychiatrist conducted interviews with the patient and families at an inpatient service unit. Final diagnosis was made by a senior psychiatrist based on ICD-10 criteria and was recorded in both inpatient medical records and the database of the ‘686’ Program.

### Measures of burden

#### Household economic burden

Current household economic burden due to schizophrenia was collected during on-site interviews in 2012 (T-study). The data collection form used was developed based on definitions of household economic indicators and standard practice in disease burden evaluations, with technical guidance from senior experts in healthcare economics. During the interview, family caregivers were asked to recall past year financial information in detail. This consisted of direct household expenses, including health expenditure, income of each family member, average time spent caregiving for the patient for each caregiver and, when applicable, the average hours patients worked each week. To facilitate accurate data collection, researchers first communicated the importance of a valid investigation, and objectively observed household living conditions. Then, expenditures, loans and balances were carefully discussed, followed by a discussion of family income.

Both direct and indirect burden were assessed and calculated in accordance with standard procedures in the field (Jan *et al*., 2018). Direct burden was defined as expenses directly related to prevention and treatment of schizophrenia, including medical expenditures (clinic visits, hospitalisation, procedures, medication, rehabilitation, etc.) and non-medical expenditures (transportation to and from the healthcare facilities, food and housing directly related to the utilisation of mental health services) in the past year.

Indirect burden consisted of the following three components: (1) Burden due to loss of patient productivity, calculated under the assumption of a 40 h work week by the formula: (average annual income in local rural individuals) × [(40) − (hours worked weekly)]/40; (2) Loss of income in caregivers due to unpaid caregiving time, calculated by the formula: (average annual income in local rural individuals) × (hours spent caregiving weekly)/40; and (3) Costs incurred by disruptive behaviour of the patient, including property damage and medical costs for physical injuries caused by the patient.

#### Caregiver burden ratings

Subjective caregiving burden was measured to better understand the impact of the disease on the daily lives of affected families. Five domains of caregiver burden were assessed: economic burden, stigma, psychological pressures, personal energy and interpersonal relationships. Each domain was rated on a 10-point scale, with ‘0’ representing ‘no burden’ and ‘10’ representing ‘extremely high burden’. Interviewees were asked to report current (T-study) burden as well as retrospectively recall burden at T-unlocking.

### Clinical measures

The following data were collected for T-study through on-site interviews and T-unlocking through reviewing patient records.

#### Medication usage

Medication usage over the previous month was collected as a categorical variable. ‘Uses medication as prescribed’ was defined as taking antipsychotics regularly and at full prescribed doses; ‘Uses medication less than prescribed’ was defined as taking medication intermittently or at doses lower than prescribed; and ‘No medication’ was defined as not taking any medication for longer than 2 weeks, regardless of whether this was due to non-compliance or because no medication was prescribed.

#### Disease severity

Global severity of disease was measured using the Clinical Global Impression – Severity (CGI-S). The scale requires clinicians to rate illness severity relative to past experiences with patients of the same diagnosis. Due to limited data in each bin, the final variable used in the analysis was grouped into three categories, where 0 = ‘Normal, not at all ill’ or ‘Borderline mentally ill’; 1 = ‘Mildly ill’ or ‘Moderately ill’; and 2 = ‘Markedly ill’, ‘Severely ill’ or ‘Among the most extremely ill patients’.

#### Violent behaviour

Violent behaviour was evaluated using a scale established during implementation of the ‘686’ Program, defined as follows: 0  =  no violent behaviour; 1  =  verbal threats or shouting but no physical violence; 2  =  destruction of property, limited to home, can be dissuaded; 3  =  destruction of property, disregarding setting, cannot be dissuaded; 4  =  repeated destruction of property or harm to people, disregarding setting, cannot be dissuaded; 5  =  use of weapons to harm property or people, arson, setting off explosions, etc., disregarding setting. In the ‘686’ Program, level 3 and above is regarded as high violence. The variable ‘significant violence’ in our analysis was defined as such.

#### Psychosocial function

Five domains of patient functioning were assessed: self-care, household chores, work and productivity, learning, and social relationships. Each domain was assessed on a three-point scale, where 0  =  poor or no function, 1  =  moderate function and 2  =  good function. The assessments were based on clinician observation of the patient's performance and reports from patients and caregivers. During data analysis, a composite functioning score was calculated by adding the scores of all five domains. In the regression analysis, the quartiles of this score were used.

### Data analysis

Data entry and management was conducted using Epidata 3.0 software with double entry. Data analysis was conducted using Stata 15.0. Wilcoxon matched-pairs signed-rank tests were used for comparison of outcomes before and after unlocking. In the risk factor analysis, univariate regression models were used to explore unadjusted effects. Multivariate regression models were built using results from the univariate analysis, supplemented by information from backwards stepwise regression. Likelihood ratio tests and Akaike Information Criteria were used to determine the final model. In the regression analysis, individuals with an indirect burden greater than CNY24 818 (*n*  =  5) were excluded based on Tukey's criteria for extreme outliers. Two subjects were excluded due to missing data. The final model included 257 subjects. All inferences were made under a significance level of *α* = 0.05.

## Results

A total of 264 patients were included, consisting of 31, 30 and 39% from Hebei, Sichuan and Guangdong province, respectively. Demographic information is presented in Table 1. The study population was predominantly male (71%) and from rural communities (98%). Mean age at the time of study was 39.6 years old. By the time of the study, most patients had been enrolled in government health insurance programmes. The average length of illness was 16.7 years.

**Table 1.** Summary of demographic and clinical characteristics (*N*  =  264)

Item	Summary measure
Demographic information	
Age, years (s.d.)	39.6 (9.1)
Female, %	29
Years of education, years (s.d.)	7.2 (3.1)
Rural residence, %	98
Unmarried, %	58
Insurance status^a^, %	
NCMS	93
URBMI	3
Other	1
No insurance	3
Clinical characteristics	
Length of disease, years (s.d.)	16.7 (7.9)
Positive family history, %	31

a Chinese citizens primarily receive public insurance. Insurance status is categorised according to the type of social health insurance scheme the individual receives. NCM  =  New Cooperative Medical Scheme, the primary insurance scheme for rural residents (individuals living in rural villages). URBMI  =  Urban Resident-based Basic Medical Insurance scheme, the primary insurance for urban residents. Other schemes include the government insurance scheme provided for employees of state-owned enterprises, and the government subsidies from the 686 Program and other publicly funded programmes. None of the participants had an urban Employee-based Basic Medical Insurance scheme (UEBMI).

Circumstances surrounding the use of restraints are presented in Table 2. At the time of the study, median time since the release was 3.2 years. Nineteen per cent of patients had been put under lock multiple times prior to ‘unlocking and treatment’. The median total time spent under restraint was 2.0 years. Managing dangerous behaviours (93%) and financial reasons (77%) were the two most commonly endorsed motives for use of restraints. The most common method of restraint was isolating patients without direct binding (59%), followed by direct binding with metal chains (33%). After participation in the intervention, 85% of subjects continued to receive mental health services. At the time of this study, 20% had experienced re-locking since being released.

**Table 2.** Description of circumstances around restraints (*N*  =  264)

Item	Summary measure
Before being freed by ‘unlocking and treatment’ intervention	
Number of times locked, %	
1	81
2–4	12
5 or more	7
Length of first restraint, years (IQR)	1.3 (0.4–6.5)
Total time under restraint, years (IQR)	2.0 (0.6–6.9)
Main reasons for restraint^a^, %	
To manage dangerous behaviours	93
Financial difficulties	77
Lack of knowledge of mental illness	34
Lack of confidence in treatment	23
Primary method of restraint, %	
Isolated in room or yard without direct binding	59
Direct binding with metal chains	33
Iron cage used	5
Direct binding with ropes	3
Settings ever used for restraint^a^, %	
Patient's own room	55
Other room in home	28
Specially built dwelling	14
Unsheltered/semi-sheltered outside of house	3
After being freed by ‘unlocking and treatment’ intervention	
Time between release and study, years (IQR)	3.2 (1.9–4.5)
Type of service used after hospitalisation, %	
Voluntary outpatient	49
Active community follow-up	34
Discontinue formal treatment	15
Long-term institutionalisation	3
Number of times relocked, %	
0	80
1	15
2 or more	5
Total time under lock if relocked^b^, years (IQR)	1.7 (0.4–3.2)

a Participants were asked to select all that applied. For some questions, responses with very low frequencies were omitted.

b Calculated among those who were relocked after T-unlocking.

After being enrolled in the ‘unlocking and treatment’ programme, we saw higher rates of medication use, decreased disease severity and reduced rates of violent behaviour (Table 3). Prior to unlocking, few subjects were able to function in any of the five domains measured, whereas by the time of this study, patient functioning improved greatly. Also, a small number of subjects (6.8%) were now economically self-sufficient. Subjective economic burden for family caregivers decreased (8.6 *v.* 6.7, *p* < 0.001), and there was a significant reduction across all the other domains of perceived caregiving burden.

**Table 3.** Changes in clinical measures and perceived caregiving burden (*N*  =  264)

Item	T-unlocking	T-study	*P* value
Medication usage, %			
Uses medication as prescribed	4	70	<0.001
Use medication less than prescribed	39	14	
No medication	56	13	
Disease severity, %			
Normal or borderline ill	0	26	<0.001
Mildly or moderately ill	2	53	
Markedly to most extremely ill	98	21	
Presence of significant violence^a^, %	66	13	<0.001
Patient able to function^b^, %			
Self-care	5	70	<0.001
Household chores	2	57	
Work and productivity	1	25	
Learning	3	22	
Social relationships	3	32	
Composite functioning score^c^, median (IQR)	0 (0–0)	2 (0–4)	<0.001
Main source of patient's finance, %			
Self-sufficient	0	7	<0.001
Family members	91	76	
Government subsidies	9	17	
Other	0	0	
Caregiver burden ratings, mean (s.d.)^d^			
Economic burden	8.6 (1.7)	6.3 (2.7)	<0.001
Stigma	7.5 (2.6)	4.7 (2.7)	<0.001
Psychological pressures	8.5 (1.8)	5.8 (2.6)	<0.001
Loss of personal energy	8.1 (2.0)	5.3 (2.6)	<0.001
Interpersonal relationship disturbance	6.9 (2.8)	4.7 (2.8)	<0.001

a Presence of significant violence was defined as violent behaviour score greater than or equal to level 3.

b Good or moderate functioning (score =  2 or 1) was coded as able to function.

c Composite functioning score was calculated by adding the scores of all domains. This composite score ranges from 0 to 10, with 0 being poor or no function in any domain, and 10 being good functioning across all domains.

d Answers were marked on a scale of 0–10, 0 being no burden, 10 being the highest burden.

The primary outcome of interest in the study was household economic burden due to disease (Table 4). Overall economic burden was estimated to be CNY12 687 (US$2004), which was substantial compared to the average family income. Indirect burden constituted the bulk of total economic burden, with a mean of CNY11 724 (US$1852) per year. Loss of productivity from both patients and caregivers were the main components of indirect burden. On the other hand, direct economic burden due to schizophrenia was only CNY963 (US$152) per year on average.

**Table 4.** Past year household economic figures (*N*  =  264)

Item	Summary statistic CNY, mean (s.d.)
Total family income	12 108 (9831)
Total family expenditure	11 641 (10 382)
Total household economic burden due to schizophrenia	12 687 (5167)
Direct economic burden	963 (1950)
Indirect economic burden	11 724 (4915)
Cost for patient loss of productivity	6806 (2545)
Cost for caregiver loss of productivity	4710 (3920)
Costs incurred by disruptive behaviour	207 (961)

Regression analysis was conducted to identify factors related to indirect economic burden (Table 5). After adjusting for other factors, residence in Sichuan province and better patient functioning at T-study were associated with a lower indirect burden. Greater disease severity at the time of study was associated with increased burden. Compared with those undergoing active community follow-up, patients who were using voluntary outpatient services, who discontinued formal treatment and who were under long-term institutionalisation had lower economic burden due to disease.

**Table 5.** Independent factors for indirect economic burden (*N*  =  257)

	Risk difference (95% confidence interval)		
Variable	Unadjusted	Full model	Final model
Constant	NA	13 141	12 891
Gender (female)	701 (−399, 1801)	907 (−85, 1900)	921 (−20, 1861)
Age (years)	62 (7, 117)	−19 (−93, 55)	
Ever married	−644 (−1767, 479)	−135 (−1255, 985)	
Province			
Hebei	REF	REF	REF
Sichuan	−1764 (−2983, −544)	−1908 (−3350, −467)	−2129 (−3414, −844)
Guangdong	1576 (429, 2723)	1090 (−295, 2477)	1012 (−149, 2173)
Education			
<6 years	REF	REF	
6–9 years	−1026 (−2337, 285)	−544 (−1665, 577)	
9–12 years	−1224 (−2535, 87)	−261 (−1381, 859)	
⩾12 years	−1347 (−3117, 423)	−704 (−2231, 823)	
Positive family history	−1070 (−2171, 31)	−782 (−1737, 174)	−882 (−1798, 34)
Length of disease, years	121 (59, 183)	65 (−15, 146)	
Time under restraint before unlocking, years	121 (28, 213)	−16 (−103, 72)	
Time between unlocking and study, years	218 (−44, 480)	−32 (−296, 232)	
Presence of relocking	419 (61, 776)	110 (−216, 436)	
Therapeutic response upon discharging from hospitalisation			
Significant improvement	REF	REF	
Improvement	−1506 (−2946, −67)	2 (−1350, 1354)	
Little to no improvement	−3611 (−5320, −1901)	−1109 (−2721, 502)	
Type of service used after hospitalisation			
Active community follow-up	REF	REF	REF
Voluntary outpatient	−1481 (−2597, −366)	−1991 (−3159, −823)	−2115 (−3179, −1051)
Discontinue formal treatment	−96 (−1654, 1463)	−1190 (−3032, 652)	−2589 (−4211, −967)
Long term institutionalisation	−2639 (−5784, 505)	−3382 (−6137, −626)	−3664 (−6296, −1032)
Medication usage at T-study			
Uses medication as prescribed	REF	REF	
Use medication less than prescribed	147 (−1218, 1513)	−193 (−1457, 1070)	
No medication	524 (−995, 2042)	−2804 (−4815, −794)	
Disease severity at T- study			
Normal or borderline ill	REF	REF	REF
Mildly or moderately ill	3204 (2086, 4321)	1327 (140, 2515)	1683 (536, 2830)
Markedly to most extremely ill	4118 (2754, 5482)	2739 (762, 4716)	2706 (850, 4563)
Significant violence at T-study	2007 (515, 3500)	1182 (−332, 2697)	
Composite functioning score at T-study			
1^st^ quartile (worst function)	REF	REF	REF
2^nd^ quartile	−694 (−1931, 543)	−559 (−1971, 851)	−706 (−2010, 599)
3^rd^ quartile	−1527 (−2805, −250)	−1036 (−2543, 470)	−1184 (−2567, 200)
4^th^ quartile (best function)	−4737 (−6064, −3409)	−3390 (−5084, −1696)	−3672 (−5238, −2107)

Risk factors for analysis were chosen based on the conceptual model, results from univariate analysis and backwards stepwise regression. The final model was determined based on likelihood ratio tests and comparison of Akaike information criteria.

## Discussion

The ‘unlocking and treatment’ programme in China is the largest venture of its kind to be reported in the scientific literature so far. Previously, reports of an intervention in Indonesia have also shown success, but it was not scaled up to the national level (Puteh *et al*., 2011; Laila *et al*., 2019). For many patients in China who were previously kept under restraints, this was the first time they could access effective, affordable and sustainable treatment. In our patient population, 85% received continuous medical care after being unlocked, and 80% were never put under lock again. In parallel with the improvement in patient mental health status and functioning, families reported significantly decreased caregiving burden.

We believe that success in unlocking patients on such a large scale can be attributable to the programme's comprehensive approach towards the major factors that led to restraining. The programme's emphasis on treatment ensured that management of psychiatric illness was a priority, which is reflected in the decrease from 65.9 to 12.8% in the proportion of patients exhibiting highly violent behaviour, and the improvement in the overall CGI-S score. Subsidies for medication and mental health services ensured that cost was not a barrier to treatment, which was reflected in the relatively low direct healthcare expenditure. The proactive nature of this programme, where community healthcare workers actively reached out to families upon learning of potential restraining, minimised barriers to care. These promising results show that the combination of active outreach and adequate financial support for treatment can successfully breach the treatment gap in this vulnerable population.

Because financial difficulty was a major reason for putting patients under restraints (Guan *et al*., 2015; Laila *et al*., 2018), understanding household economic burden in this population is crucial for preventing future cases of restraining. Zhai *et al*. studied the economic burden due to schizophrenia in patients treated at two leading psychiatric hospitals in China (Zhai *et al*., 2013). The indirect costs in their study averaged US$1723.4 (CNY10 685), which is comparable to our mean of CNY11 724 per year. However, in their study, the direct costs of schizophrenia were on average US$862.8 (CNY5349 using 2013 conversion rates), over fivefold higher than the CNY963 found in our study. Previous reports from Thailand and Ghana also found higher proportions of direct financial burden at 39 and 18%, respectively (Phanthunane *et al*., 2012; Opoku-Boateng *et al*., 2017), compared to only 2% in our study. This is largely due to government policies to reduce healthcare costs in China, including health insurance coverage and additional treatment subsidies for the underprivileged through the ‘686’ Program. As the treatment centres in the study of Zhai *et al*. were urban hospitals affiliated to medical universities (Zhai *et al*., 2013), their study population were likely of higher socioeconomic status and thus not eligible for such subsidies.

Loss of patient capacity to work was a major contributor to indirect economic burden, closely followed by loss of caregiver productivity due to unpaid caregiving time. It has been suggested that policies regarding non-communicable diseases need to look beyond the out-of-pocket costs of healthcare (Jan *et al*., 2018). As the ‘686’ Program continues to develop, future amendments should emphasise the provision of occupational and psychosocial support for both patients and caregivers, as well as exploring methods to account for unpaid caregiving time. This has been implicated in a new national pilot programme launched in 2015 (Zhang and Ma, 2017).

Results of the multivariate analysis of risk factors showed that patient disease severity and psychosocial functioning at the time of study were independently associated with indirect economic burden. The relationship between these two factors and indirect burden both follow a dose–response pattern, suggesting potential causality. This finding can assist in identifying high-risk groups who may benefit from intensive psychosocial assistance aimed at reducing financial burden. Furthermore, our results attest to the beneficial value of achieving good treatment outcomes in improving the lives of affected individuals and their families.

Our results also showed that those undergoing active community follow-up had a greater indirect burden compared to other service utilisation methods. This is possibly due to confounding by indication, as families deemed to be in greater need of assistance were preferably considered for active follow-up. Patients under active follow-up in our study were on average older (42 *v.* 38 years), and more prone to violent behaviour before unlocking (*p* values <0.001). Caregiving for patients with higher risk of violence is likely more time consuming, which may result in greater loss of productivity. Individuals of higher age are less competitive in the labour market, and thus bring in less income. More investigation is warranted to understand the underlying reasons for this complicated financial phenomenon.

### Limitations

Our ability to directly compare economic burden before and after participation in the ‘unlocking and treatment’ intervention was limited, as we were unable to acquire financial information prior to unlocking. We hope that the results from this study will incentivise enrichment of baseline economic data collection in future programme implementation. Another limitation is that participants might be reluctant to disclose accurate economic information due to concerns about future service provision. To address this issue, the key researchers responsible for collecting data were the city or provincial-level psychiatric professionals rather than community health workers, and thus were not direct service providers. Furthermore, interviewers would reassure patients that information was collected only for research purposes. Objective observations were used to further verify economic information.

We acknowledge that retrospectively measuring family subjective burden at T-unlocking may introduce recall bias. Therefore, we did not analyse these data in depth during the final regression analyses. Putting a family member under restraints and their subsequent release were extremely significant life events, which likely remained poignant in caregivers' memories, and these data were presented to reflect this subjective experience.

### Implications

This study addresses the crisis of chronic restraining of the mentally ill in low-resource settings. As financial difficulties are a major trigger for restraining patients, understanding household economic burden and its related factors is crucial to unravelling this phenomenon. Our study describes an intervention that demonstrates promising outcomes, and furthermore highlights the importance of improving patient functioning and addressing unpaid caregiving time in reducing financial burden. Hopefully, culminating work in this field will lead to the eventual eradication of restraints and bring forth a world where the fundamental rights to health and autonomy of patients with mental disorders are fully recognised.

## Availability of data and materials

Data can be made available upon request. However, sharing of the data may require IRB approval and some access restrictions may apply. Requests may be sent to the corresponding author.
